# Quantification of HPV16 E6/E7 mRNA Spliced Isoforms Viral Load as a Novel Diagnostic Tool for Improving Cervical Cancer Screening

**DOI:** 10.3390/jcm7120530

**Published:** 2018-12-08

**Authors:** Claire Camus, Sébastien Vitale, Céline Loubatier, Guillaume Pénaranda, Hacène Khiri, Anne Plauzolles, Xavier Carcopino, Philippe Halfon, Valérie Giordanengo

**Affiliations:** 1 Clinical Research and R&D Department, Laboratoire Européen Alphabio, 13003 Marseille, France; g.penaranda@alphabio.fr (G.P.); h.khiri@alphabio.fr (H.K.); a.plauzolles@alphabio.fr (A.P.); philippe.halfon@alphabio.fr (P.H.); 2 Department of Virology, Biological and Pathological Center, Centre Hospitalier Universitaire de Nice, 06200 Nice, France; vitale.s@chu-nice.fr (S.V.); Celine.LOUBATIER@unice.fr (C.L.); Valerie.Giordanengo@unice.fr (V.G.); 3 Institut National de la Santé et de la Recherche Médicale (INSERM), 06200 Nice, France; 4 Faculty of Medicine, Côte d’Azur University (UCA), 06107 Nice, France; 5 Department of Obstetrics and Gynaecology, Hôpital Nord, APHM, Aix-Marseille University (AMU), Univ Avignon, 13000 Marseille, France; xcarco@free.fr; 6 Infectious and Internal Medicine Department, Hôpital Européen Marseille, 13003 Marseille, France

**Keywords:** high risk human papillomaviruses (HPVs) infection, cervical cancer, HPV16 E6/E7 mRNA viral loads, qRT-PCR, diagnostic tool, grade 2 or higher cervical intraepithelial neoplasia

## Abstract

High-risk human papillomaviruses (HPVs) have been identified as the main contributors to cervical cancer. Despite various diagnostic tools available, including the predominant Papanicolaou test (Pap test), technical limitations affect the efficiency of cervical cancer screening. The aim of this study was to evaluate the diagnostic performance of spliced HPV16 E6/E7 mRNA viral loads (VL) for grade 2 or higher cervical intraepithelial neoplasia diagnosis. A new dedicated (quantitative reverse transcription polymerase chain reaction) qRT-PCR assay was developed, allowing selective quantification of several HPV16 E6/E7 mRNA: Full length (FL) with or without all or selected spliced forms (total E6/E7 mRNA corresponding to SP + E6^E7 mRNA (T), + spliced E6/E7 mRNA containing intact E7 ORF (SP), and E6/E7 mRNA containing disrupted E6 and E7 ORFs calculated by the following subtraction T-SP (E6^E7)). Twenty HPV16 DNA and mRNA positive uterine cervical smears representative of all cytological and histological stages of severity were tested. We have shown that all E6/E7 mRNA isoforms expression levels were significantly increased in high grade cervical lesions. Statistical analysis demonstrated that the SP-E6/E7 VL assay exhibited: (i) The best diagnostic performance for identification of both cervical intraepithelial neoplasia (CIN)2+ (90% (56–100) sensitivity and specificity) and CIN3+ (100% (72–100) sensitivity and 79% (49–95) specificity) lesions; (ii) a greater sensitivity compared to the Pap test for CIN2+ lesions detection (80% (44–97)); (iii) a predictive value of the histological grade of cervical lesions in 67% of atypical squamous cells of unknown significance (ASC-US) and 100% of low-grade (LSIL) patients. Overall, these results highlight the value of SP-E6/E7 mRNA VL as an innovative tool for improving cervical cancer screening.

## 1. Introduction

Cervical cancer (CC) is the second most common cancer among women worldwide, with more than 528,000 new cases in 2012 [1]. The causative role of human papillomaviruses (HPVs) in CC has been well documented, and epidemiological data showed that 99.7% of invasive CC cases were associated with positive detection of HPV [2,3]. Other types of malignancies such as vaginal, vulvar, penile, anal, or oropharyngeal cancers have been associated with HPV [4]. More than 150 HPV types have been identified so far and are classified as high-risk (HR-HPV) or low-risk of developing cancer, depending on their transforming ability. Forty HPV types were associated with genital tract infection, thirteen of which were classified as oncogenic HR-HPV viruses [5,6,7]. Meta-analysis studies and a recent international large retrospective study based on more than 10,000 invasive CC histological-confirmed cases established the predominant involvement of 8 HR-HPV types in invasive CC: 16, 18, 45, 33, 31, 52, 58, and 35, ranked by decreasing prevalence [8,9]. Moreover, International Agency for Research on Cancer (IARC) and World Health Organization (WHO) institutions recognized the unique carcinogenic strength of HPV16 [10].

HPV infection of the cervix is usually transient, asymptomatic and cleared spontaneously within 9 to 15 months [11]. In a small fraction of women, HR-HPV infection may persist, resulting in the development of precancerous lesions, defined as cervical intraepithelial neoplasia (CIN), which may progress to invasive CC [12]. Currently, CC screening relies essentially on the liquid-based cytology Papanicolaou test (Pap test), allowing the detection of abnormal neoplastic cells in cervical scrapes [13]. The implementation of a systematic Pap test, as well as improvements in CIN clinical management therapeutics have already proven to be successful in reducing the incidence of CC-induced mortality [14,15]. However, this test shows several limitations, including a low clinical sensitivity, often resulting in delayed diagnosis of cervical lesions [16]. In France, 30% of CC occurs in less than three years following a normal cytological Pap test [17]. There is an urgent need to improve cervical cancer screening to enable early diagnosis and improve the prognosis of CC [18].

HR-HPV DNA testing detects early HR-HPV infections in uterine cervical samples (UCS) for the identification of patients at risk of developing cervical lesions. It has recently been introduced as a complement to the Pap test screening for women over 30 years old [19]. The HR-HPV DNA test exhibits greater sensitivity than cytological analysis but at the expense of the specificity, resulting in over-management of women with a positive test [20]. Nevertheless, the improved diagnostic performance of HR-HPV DNA testing over cytology has led healthcare authorities to promote the implementation of this test for CC primary screening. In 2014, United States Food and Drug Administration approved the first HPV DNA assay for primary CC screening. In France, HR-HPV testing is recommended only for two clinical conditions: Follow-up of ambiguous cervical cytological smear with atypical squamous cells of unknown significance (ASC-US) status and post-conization monitoring. French Health Authorities have announced a future switch to a Pap test-based organized screening with a call-recall system for non-participating women every three years. This organized screening could pave the way for the implementation of HR-HPV DNA tests.

Given the current limitations of CC screening tests, there is a real need to develop new diagnostic assays with greater and well-balanced Sensitivity-Specificity scores, using new biomarker candidates related to CC pathogenesis [21,22]. Based on previous data suggesting that HR-HPV DNA integration into the host cell genome is an essential step to malignant transformation, plenty of studies have investigated the relationships between HR-HPV physical status and occurrence of cervical lesions [23,24,25]. The screening value of HR-HPV DNA viral load (VL) as a marker of the extent of HPV infection has also been assessed [26,27].

Another promising candidate for early diagnosis of HR-HPV and especially HPV16-associated cervical lesions is HPV E6/E7 mRNA [28]. E6 and E7 expression results from the transcription of a bicistronic pre-mRNA, which is further spliced to generate several mRNA isoforms able to selectively produce functional or truncated proteins. Ajiro et al. identified three 5′ splicing sites and three 3′ splicing sites, all used in CC tissues and cell lines to generate six different mRNA species [29]. Some of the E6 and E7 truncated isoforms such as E6*I and (E6/E7 mRNA containing disrupted E6 and E7 ORFs calculated by the following subtraction T-SP) E6^E7, regulate E6 and E7 expression, induce trans-activation of the P97 viral promoter, and/or stabilize both proteins leading to an increase in their half-life [30,31].

Indeed, it is well accepted that an increase in E6/E7 mRNA and derived oncogenic proteins is a hallmark of the progression from a transient to a transforming HPV infection [32]. Thus, identification of HPV infections associated with E6/E7 transcriptional activities might be more relevant than the detection of the sole presence of HPV DNA in predicting the infection outcome. Qualitative assays for detection of E6/E7 mRNA expression in UCS have been marketed. Their clinical performance evaluations have shown an increase in specificity over DNA tests with concomitant loss of sensitivity. One of the limitations of these qualitative tests is their inability to discriminate between low and high transcriptional activity infections. As HPV carcinogenesis has been linked to overexpression of E6 and E7 proteins, qualitative tests may overestimate the risk of malignant progression for a subset of patients experiencing low E6/E7 mRNA VLs. Interestingly, a previous study has shown that the HPV16 E6/E7 mRNA copy number, as measured in ThinPrep cytological samples, gradually increases with cervical lesion severity [33]. More recently, Ho et al. showed that E6/E7 mRNA load, but not viral DNA load, is correlated with the histopathologic severity of cervical lesions [34].

To explore the diagnostic performance of E6/E7 mRNA quantification, we have developed, for the first time, a quantitative reverse transcription polymerase chain reaction (qRT-PCR) assay. This test allowed a selective quantification of various HPV16 E6/E7 mRNA isoforms in UCS and characterization of the diagnostic validity for identification of high grade CIN2+ (CIN of grade 2 or more: CIN2, CIN3, cancer) and CIN3+ (CIN of grade 3 or more: CIN3, cancer) lesions. We then compared the clinical performance of this assay to the “gold standard” Pap test and to a previously described qPCR-based assay quantifying HPV16 DNA VL.

## 2. Materials and Methods

### 2.1. Cell Lines

The SiHa cell line (LGC Standards, Molsheim, France), containing 1 to 2 integrated genome copies of HPV16, was established from fragments of a primary tissue sample grade II squamous cell carcinoma surgically collected from a 55 years Japanese woman [35].

### 2.2. Patients

According to the French recommendations, cancer cervical screening should be done every 3 years and is performed using liquid-based cytological analysis performed on uterine cervical smears (UCS). UCS were obtained from 20–65 years women during routine gynecological examinations performed in collaborating centers with our laboratory. Informed consent from all 502 individuals was obtained prior to enrollment, and no donor specific identifying information was collected or used in the course of this study. This study protocol was approved by an independent ethics committee (Comité de Protection des Personnes Sud-Méditerranée II, ID-RCB 2012-A00541-42) and is consistent with international ethical standards on human subjects’ research.

### 2.3. Uterine Cervical Samples Analysis and Criteria of Selection

Cervical samples were taken from the transformation zone using a Cervex–Brush (Rovers Medical Devices, Oss, The Netherlands), placed into PreservCyt medium (Hologic, Roissy, France) women. Cytological analysis was systematically performed on each sample using the ThinPrep liquid Pap test (Hologic, Roissy, France) according to the manufacturer’s instructions. HR-HPV DNA test was performed in case of cytological abnormality and to explore the origin of clinical manifestations such as post-coital metrorrhagia or follow-up a previous HR-HPV positive test. All women presenting cervical cytological abnormalities (regardless on the abnormality severity) or HR-HPV DNA-positive test result were immediately referred for colposcopy examination. Colposcopic evaluation was considered adequate if the squamocolumnar junction was clearly visible. The slightest abnormality detected during colposcopy was biopsied. Cytology and histology results were obtained from centralized analysis performed according to the 2001 Bethesda System classification and the international nomenclature [36]. When a biopsy was performed, final diagnosis of cervical lesion severity grade was exclusively established, based on the histological analysis gold standard.

In order to evaluate cancer cervical screening clinical performances of our HPV16 DNA and mRNA VL quantification tests in comparison to the histology gold standard, we selected a representative panel of patients according to the following criteria: (i) HPV16 DNA mono-infected patient (Papillocheck^®^ Assay, Greiner Bio-One, Courtaboeuf, France) expressing E6/E7 mRNA (APTIMA^®^ HPV assay (Hologic, Roissy, France); (ii) suitability of nucleic acid extracts for molecular analysis (Thermo Scientific™ NanoDrop™ 1000 spectrophotometer, Thermo Fisher Scientific, Villebon-sur-Yvette, France); and (iii) all cytological (5 samples were graded normal, 3 ASC-US, 4 low grade squamous intraepithelial lesion (LSIL), 4 atypical squamous cells-cannot exclude high grade (ASC-H) and high grade squamous intraepithelial lesion (HSIL)) and histological (2 lesion-free samples, 8 CIN1, 4 CIN2, 4 CIN3, and 2 invasive cancers) severity grades should be represented. Twenty UCS were selected according to those previous criteria. Residual liquid from those UCS were stored at −20 °C for further virological analysis.

### 2.4. HR-HPV DNA Genotyping

Digene test positive samples were subjected to nucleic acid extraction on the NucliSENS^®^ easyMAG^®^ platform (bioMérieux, Craponne, France), and tested with the Papillocheck^®^ Assay (Greiner Bio-One, Courtaboeuf, France) according to the manufacturer’s instructions. The PapilloCheck^®^ HPV assay is a polymerase chain reaction (PCR) based assay designed to detect and genotype by DNA chip technology 24 HPV types including 18 HR or possibly HR types (16, 18, 31, 33, 35, 39, 45, 51, 52, 53, 56, 58, 59, 66, 68, 70, 73, 82) plus 6 low-risk types (6, 11, 40, 42, 43, 44).

### 2.5. HR-HPV E6/E7 mRNA Detection

Nucleic acid extracts from women mono-infected with HPV16 were analyzed using the APTIMA^®^ HPV assay (Hologic, Roissy, France) according to the manufacturer’s instructions. This test allows qualitative detection (without genotyping) of HPV E6/E7 mRNA from 14 HR-HPV types (16, 18, 31, 33, 35, 39, 45, 51, 52, 56, 58, 59, 66, and 68).

### 2.6. Nucleic Acid Extractions

DNA dedicated to the HPV16 DNA VL assay was extracted from 1 mL of liquid PreservCyt specimens derived from HPV16 mono-infected patients on the NucliSENS^®^ easyMAG^®^ platform (bioMérieux, Craponne, France). Extraction was performed using a Generic 2.0.1 protocol with on-board lysis, and 50 µL of NucliSENS^®^ easyMAG^®^ magnetic silica was added for nucleic acids capture. Nucleic acid extracts were collected in 25 μL of easyMAG^®^ elution buffer.

Total RNA to be used in the HPV16 E6/E7 mRNA VLs assay was extracted from PreservCyt samples using the Masterpure™ RNA Purification Kit (Epicentre, Madison, WI, USA). UCS were centrifuged at 1800 *g* for 10 min. Pellets were washed with 5 mL of PBS, centrifuged at 2200 *g* for 10 min, and resuspended in 600 µL of lysis solution containing 540 µL “Tissue and Cell Lysis Solution” and 60 µL Proteinase K (Thermo Fisher Scientific, Villebon-sur-Yvette, France) at 50 mg/mL. Lysis solution was incubated overnight at 60 °C, and total nucleic acids were extracted using the Masterpure™ Kit following the manufacturer’s instructions. The nucleic acids pellet was resuspended in TE Buffer. Nucleic acids concentration and quality were assessed by spectrophotometry. DNA was digested by DNase I (Thermo Fisher Scientific, Villebon-sur-Yvette, France) at a ratio of 2 DNase units per µg of total nucleic acids for 15 min at room temperature. EDTA (ethylene diamine tetraacetic acid) was added at a final concentration of 2.5 mM to prevent RNA scission during DNase heat inactivation. RNA was incubated at 65 °C for 10 min, and then stored at −80 °C until needed.

### 2.7. HPV16 DNA VL Quantification Assay

The qPCR assay used for HPV16 DNA VL quantification was adapted from the method previously published by Peitsaro et al. [37]. This method allows co-amplification of E2 and E6 HPV genes for selective evaluation of total, episomal and integrated VLs. Sequences of primers and probes used to amplify HPV16 genes were identical to the Peitsaro et al. study, but amplification of the human β-globin gene was added to normalize VLs between samples (Table 1).

Primers and probes were diluted at a final concentration of 0.9 µM and 0.25 µM, respectively. UCS extracts (2.5 µL) were added to PCR reactions, along with 5 µL of 2 × TaqMan^®^ Fast Universal PCR Master Mix (Thermo Fisher Scientific, Villebon-sur-Yvette, France). Thermocycling conditions consisted of 20 s at 95 °C, followed by 40 two-step cycles at 95 °C for 1 s and 60 °C for 20 s. Amplifications were performed on an Applied Biosystems^®^ 7500 Real-Time PCR system (Thermo Fisher Scientific, Villebon-sur-Yvette, France). Data were analyzed using the 7500 software v2.0.6. E2, E6, and β-globin copy numbers in UCS were determined by absolute quantification using standard curves. These curves were generated from serial dilutions of plasmids containing E2, E6, and β-globin’s relevant sequences (ten-fold range). β-globin amplification allowed cell load quantification (copy number divided by two, as each cell contains two copies of the β-globin gene). Total and episomal HPV16 VLs were determined using E6 and E2 cell load normalized DNA quantifications. Integrated VL was calculated by subtracting episomal VL from total VL.

### 2.8. HPV16 E6/E7 mRNA VLs Quantification Assay

**Figure 1:**
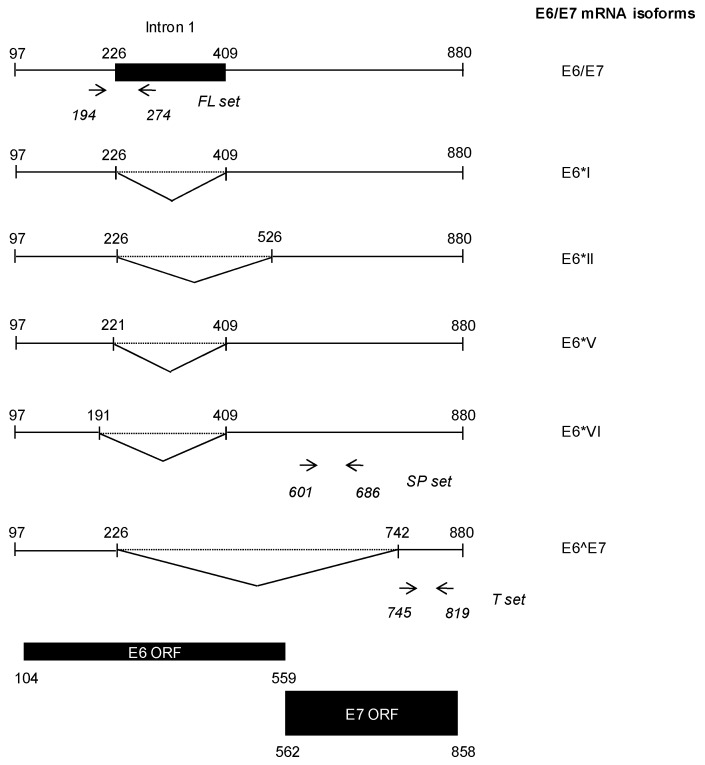
Characteristics of E6/E7 mRNA species amplified in the E6/E7 quantitative reverse transcription polymerase chain reaction (qRT-PCR) assay.

HPV16 E6/E7 mRNA isoforms were amplified and quantified using TaqMan chemistry and the SuperScript III Platinum One-Step qRT-PCR system (Thermo Fisher Scientific, Villebon-sur-Yvette, France). Three primer/probe sets were designed. The full length (FL set amplifies only unspliced transcripts, the (total E6/E7 mRNA corresponding to SP + E6^E7 mRNA) T set detects all possible isoforms and the (+ spliced E6/E7 mRNA containing intact E7 ORF) SP set targets all mRNA except the E6^E7 isoform Figure 1). The E6^E7 transcript load was calculated by subtracting the SP load from the T load. E6/E7 mRNA expression levels were normalized to β-actin as previously described in exfoliated cervical cells [38]. The primer/probe’s sequences from the different sets used are shown in Table 2.

Illustration of E6/E7 mRNA isoforms produced by alternative splicing of the E6/E7 bicistronic pre-mRNA. Utilization of the nucleotide 226 5′ splice site generates three different species (i.e., E6*I. E6*II, and E6^E7) depending on the 3′ splice site used. E6*I and E6*II display shortened E6 ORF but retain a complete E7 ORF and are thus able to produce E7 in addition to two truncated E6 proteins. In the E6^E7 mRNA isoform, both E6 and E7 ORFs are truncated, leading to translation of a E6/E7 fusion protein. Splicing through the nucleotide 409 3′ splice site results in two additional mRNA isoforms, E6*V and E6*VI, which contain truncated E6 ORFs and the full length E7 ORF. Location of the forward and reverse primers designed for the FL, SP and T primer/probe sets are indicated in italics.

Primers and probes were used at a final concentration of 200 nM and 100 nM, respectively. RNA was reverse transcribed for 5 min at 50 °C, heated at 95 °C for 2 min to inactivate reverse transcriptase, and amplified by qPCR using 40 two-step cycles at 95°C for 3 s and 60 °C for 30 s. E6/E7 and β-actin mRNA loads in UCS were quantified using the absolute quantification standard curve method. Standard curves were established by amplifying ten-fold serial dilutions produced from recombinant RNA stock solutions of known concentrations. Recombinant RNA was produced using an in vitro transcription Riboprobe^®^ T7 System (Promega, Charbonnières-les-Bains, France) following the manufacturer’s instructions. Recombinant sequences were obtained from plasmids containing E6/E7 sequences and β-actin mRNA downstream of the T7 promoter. All qRT-PCR were performed on an Applied Biosystems^®^ 7500 Real-Time PCR system (Thermo Fisher Scientific, Villebon-sur-Yvette, France), and data were analyzed using the 7500 software v2.0.6.

### 2.9. Statistical Analysis

The clinical performances of our HPV16 DNA and mRNA tests for the detection of cervical disease were determined by comparing assay results with the final diagnosis. Final diagnosis of CIN2+ and a CIN3+ were considered as primary and secondary disease endpoints.

We applied logistic regression and univariate analysis to assess diagnostic performances of HPV16 DNA VLs, E6/E7 mRNA VLs, and Pap test for identification of CIN lesions. We generated Receiver Operating Curves (ROC) and evaluated the performances of each parameter using Area Under ROC Curve (AUC), sensitivity, specificity, negative and positive predictive values determination for HPV16 DNA VLs and E6/E7 mRNA VLs parameters. In an attempt to reach the optimal combination of sensitivity and specificity, the threshold value was first established according to the highest Youden index (sensitivity + specificity − 1) to reach the maximal sensitivity and secondly to the highest specificity. Wilcoxon non parametric test was used to test statistical significance of VLs regarding proper classification of lesion grades. *p*-values ≤0.05 were considered statistically significant. Clinical performances such as sensitivity, specificity, negative predictive value (NPV), and positive predictive value (PPV) were expressed in percentages (95% confident intervals). Statistical calculations were performed using SAS V9.4 software (SAS Institute Inc., Cary, NC, USA).

## 3. Results

### 3.1. Validation of HPV16 DNA and E6/E7 mRNA VLs Quantification Assays

We first validated our qPCR assay dedicated to quantification of HPV16 DNA VLs through amplification of E2, E6, and β-globin genes in the SiHa cell line. SiHa cells have been previously described to harbor 1 to 2 copies of the HPV16integrated genome into the host cell genome [35]. For total DNA VL, a mean value of 0.93 ± 0.2 (0.7–1.06) copies/cell was obtained from three independent experiments, thus being close to the expected theoretical value (Table 3). There was no amplification of the E2 gene in SiHa cells, reflecting a complete integration of HPV16 into the host genome.

Eventually, our E6/E7 mRNA qRT-PCR assay was validated through successful amplification and quantification of E6/E7 mRNA in SiHa cells. Table 3 shows values obtained with primer/probe sets targeting various mRNA produced from splicing of the bicistronic full length E6/E7 mRNA (Figure 1).

As expected, knowing that T-E6/E7 amplifies the E6^E7 mRNA isoform in addition to mRNA species targeted by SP-E6/E7, the T-E6/E7 mRNA VL was higher than the SP-E6/E7 one (4.93 ± 0.23 (4.68–5.12) versus 4.86 ± 0.21 (4.63–5.00) log_10_ copies/10^6^ β-actin mRNA copies on three independent experiments). Moreover, FL-E6/E7, which targets only unspliced E6/E7 mRNA, yielded a VL of 4.47 ± 0.18 (4.27–4.59) log_10_ copies/10^6^ β-actin mRNA copies, which was, as anticipated, lower than SP-E6/E7 VL. We detected expression of E6^E7 mRNA in SiHa cells, yet at a lower level than FL-E6/E7 mRNA with a VL of 4.09 ± 0.37 (3.73–4.48) log_10_ copies/10^6^ β-actin mRNA copies.

### 3.2. Quantification of HPV16 DNA and E6/E7 mRNA VLs in UCS

We have selected a cytological and histological representative panel of 20 UCS derived from HPV16 mono-infected women displaying an APTIMA^®^ E6/E7 mRNA positive test. UCS were also selected according to quality of nucleic acids extracted. VLs quantification was performed on residual UCS. Table 4 shows results of cytological and histological grading, along with quantifications of HPV16 DNA and E6/E7 mRNA VLs.

Quantifications of DNA VLs in UCS showed a large range of values for total and integrated VLs, ranging from 5.35 to 8.09 log_10_ copies/10^6^ cells and from 4.81 to 7.97 log_10_ copies/10^6^ cells, respectively. Interestingly, high DNA VLs seemed to be associated with high grade lesions, as both invasive cancers and 3 out of 4 CIN3 lesions exhibited DNA VLs superior to 7 log_10_ copies/10^6^ cells. In contrast, only 2 out of 8 low grade CIN1 lesions showed DNA VLs higher than 7 log_10_ copies/10^6^ cells, and both lesion-free samples had VLs lower than 6.5 log_10_ copies/10^6^ cells. Most samples, regardless of lesion grade, displayed high levels of HPV integration as integrated VLs were higher than episomal VLs in these UCS. Surprisingly, HPV integration was low for both invasive cancer samples. Indeed, episomal VL was higher than integrated VL for patient 3, while episomal VL was barely lower than integrated VL for patient 6.

Regarding E6/E7 mRNA VL quantifications, values in log_10_ copies/10^6^ β-actin mRNA copies varied depending on the parameter measured: FL-E6/E7, SP-E6/E7, T-E6/E7, and E6^E7 range were (0–4.25), (0–5.16), (1.91–5.67), and (1.91–5.64), respectively. Interestingly, the highest E6/E7 mRNA loads were associated with high histological grade lesions (CIN3 and Cancer) for the three primer/probe sets. Indeed, all cancers and CIN3 samples, as well as three out of four CIN2 samples, had a SP-E6/E7 VL superior to 3 log_10_ copies/10^6^ β-actin mRNA copies. It should be noted that VLs measured for both invasive cancers were in the same range than those obtained for SiHa cancer cells. In contrast, only two out of eight CIN1 samples had a SP-E6/E7 VL higher than 3 log_10_ copies/10^6^ β-actin mRNA copies, while both unlesional samples had a VL lower than 3 log_10_ copies/10^6^ β-actin mRNA copies. Four samples (patients 4, 42, 44, and 51) showed undetectable SP-E6/E7 mRNA load while two of them (patients 4 and 44) did not express FL-E6/E7 mRNA, an unexpected amplification of these unspliced mRNA was detectable in patients 42 and 51. Surprisingly, despite exhibiting a CIN2 lesion at histological examination, patient 4’s cervical smear only contained E6^E7 mRNA, as amplification with the T-E6/E7 primer/probe set was positive. As for the other E6/E7 mRNA VLs, the E6^E7 load appeared to correlate with the lesion severity grade.

### 3.3. HPV16 E6/E7 mRNA VLs Are Increased in High Grade Cervical Lesions

Univariate analysis was used to assess the statistical significance of the increase in HPV16 DNA and E6/E7 mRNA VLs observed in UCS derived from patients suffering from CIN2+ and CIN3+ lesions. We calculated the arithmetic means of DNA and E6/E7 mRNA VLs for two paired groups of patients: <CIN2 versus CIN2+ and <CIN3 versus CIN3+. The Wilcoxon rank-sum test was then used to compare the ability of DNA VLs and E6/E7 mRNA VLs quantifications to identify properly high grade CIN2+ or CIN3+ lesions.

Results showed that DNA and mRNA VLs arithmetic means were increased in high grade lesions, for both CIN2 and CIN3 thresholds (Table 5). This increase was statistically significant for each E6/E7 mRNA VL regardless of both histologic thresholds (*p*-Value < 0.05). Regarding DNA VLs, only the total DNA VL increase was statistically significant for CIN3+ lesions. These data suggest that E6/E7 mRNA VLs are relevant biomarkers to discriminate patients harboring high grade cervical lesions from patients with low grade lesions regardless of high or low E6/E7 mRNA expression level.

### 3.4. Comparison of the Pap Test, HPV16 DNA VLs, and HPV16 E6/E7 mRNA VLs Sets Diagnostic Performances for Detection of High Grade Cervical Lesions

**Figure 2:**
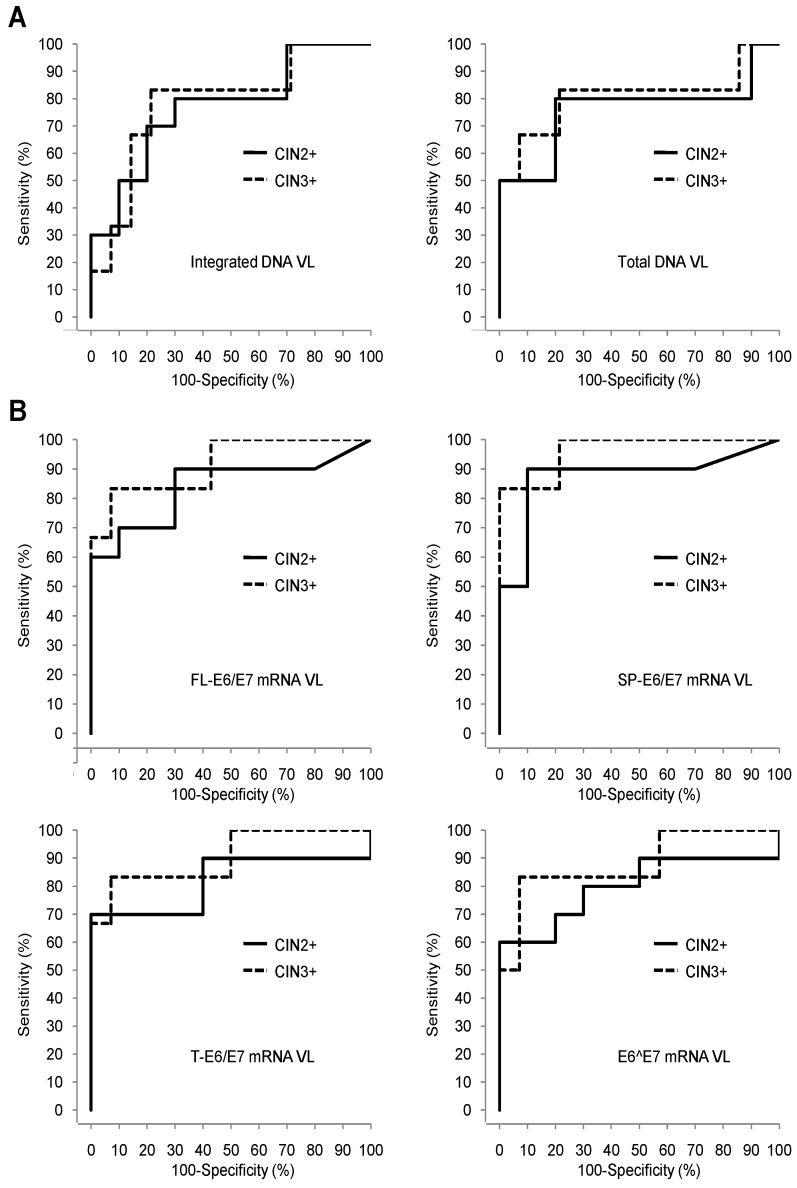
Receiver Operating Characteristic (ROC) curve analysis of human papillomavirus (HPV) 16 DNA and E6/E7 mRNA viral load (VL) quantification assays for classification of CIN2+ and CIN3+ lesions. (**A**) ROC curves obtained from analysis of integrated (left-hand panel) and total (right-hand panel) DNA VL data. (**B**) ROC curves obtained from analysis of FL-E6/E7 (top left-hand panel). SP-E6/E7 (top right-hand panel). T-E6/E7 (bottom left-hand panel) and E6^E7 (bottom right-hand panel) mRNA VL data.

In order to measure the effectiveness of HPV16 DNA and E6/E7 mRNA VLs quantification assays for proper identification of high grade CIN2+ and CIN3+ cervical lesions, we performed a ROC analysis. ROC curves obtained for detection of CIN2+and CIN3+ lesions are shown in Figure 2A for DNA VLs and Figure 2B for E6/E7 mRNA VLs. The AUC value calculated for each VL reflects its accuracy in discriminating high grade lesions from low grade ones.

For both CIN2+ and CIN3+ lesions, quantification of SP-E6/E7 mRNA VL exhibited the best performance with an AUC of 0.88 (95% CI, 0.69–1.00) and 0.97 (0.89–1.00), respectively (Table 6). The remaining three E6/E7 mRNA VLs, as well as DNA VLs, displayed lower AUC.

We estimated optimal diagnostic thresholds for DNA and E6/E7 mRNA VLs defined as the cut-off values resulting in the highest Youden Index (YI) associated with primarily maximized sensitivity and secondarily maximized specificity (Table 6). The best diagnostic performance was observed for SP-E6/E7 VL with thresholds set at 3.16 and 3.17 log10 copies/10^6^ β-actin mRNA copies for CIN2+ and CIN3+, respectively. These thresholds were associated with the highest sensitivity/specificity balance (90%–90% (56–100)) and YI of 0.80 for CIN2+ lesions. On the other hand, while the optimal diagnostic thresholds calculated for FL-E6/E7, T-E6/E7 and E6^E7 VLs also resulted in 90% sensitivity, the specificity and YI were decreased. DNA VLs performances were lower than that of SP-E6/E7 VL, as optimal diagnostic thresholds calculated allowed to reach a maximal sensitivity of 80% (44–97) for CIN2+ lesions. For CIN3+ lesions, the Sensitivity/Specificity balance was 100% (54–100)–79% (49–95) for SP-E6/E7 VL and the YI was 0.79. As observed for CIN2+ lesions, FL-E6/E7, and E6^E7 VLs were associated with reduced specificity, while T-E6/E7 and DNA VLs exhibited decreased sensitivity.

We also assessed the diagnostic performance of the Pap test, defined as the clinical “gold standard”, in our cohort. For this purpose, we considered that UCS exhibiting normal, LSIL or ASC-US cytology results were predictive of low grade histological lesions, while samples classified under the ASC-H or HSIL categories were indicative of the presence of a high grade lesion. This discrimination is based on epidemiological studies showing that CIN2+ lesions are found in only 5% to 10% of ASC-US patients, while 40% of ASC-H UCS are associated with a CIN2+ lesion [39]. 

According to this classification, we obtained a sensitivity of 80% (44–97) for the Pap test for identification of CIN2+ lesions, which was lower than the one observed for SP-E6/E7 mRNA VL (90% (56–100)) (Table 6). The Pap test exhibited higher specificity and PPV than SP-E6/E7 mRNA VL (100% (63–100) vs. 90% (56–100)), but a lower NPV (83% (52–98) vs. 90% (56–100)). For CIN3+ lesions, sensitivity (100% (54–100)) and NPV (100% (72–100)) reached the highest possible value for both the Pap test and SP-E6/E7 mRNA VL, while specificity (86% (57–98) vs. 79% (49–95)) and PPV (75% (35–97) vs. 67% (30–93)) were slightly better for the Pap test.

## 4. Discussion

One of the biggest challenges in the area of HPV is improving CC testing. Despite the introduction of the Pap test more than 50 years ago and the recent development of HPV DNA tests, CC remains a worldwide public health issue [39]. This is partly due to the limitations of the currently used CC screening tests, which display insufficient sensitivity and/or specificity for the detection of cervical lesions [40]. Several biomarkers of host cell infection or targeting viral components have been proposed and studied to optimize CC screening [22]. Among them, the expression E6/E7 mRNA has been associated with the progression of CC. E6/E7 mRNA detection tests exhibit good clinical performances for the identification of high grade cervical lesions [41]. In this study, we developed an E6/E7 mRNA quantification test in order to improve proper detection of CIN2+ or CIN3+ and consequently of CC. 

First, quantification of DNA VLs in our UCS cohort shows that both integrated and total VLs levels are generally increased in women suffering from high grade cervical lesions, albeit in a non-statistically significant manner (Table 5). HPV16 DNA VLs were quantified using a real-time PCR assay as previously described by Peitsaro et al. [37]. Our results were consistent with this study showing that highly integrated LVs are associated with high grade CIN lesions, which reinforces that it may be a marker of poor prognosis for lesion progression. As shown by Peitsaro et al., we also found that most of the samples, including UCS derived from non-lesional and CIN1 cervixes, contained both integrated and episomal HPV16 viruses. These results indicate that HPV integration into the host genome is an early event in HPV-induced cervical carcinogenesis. Surprisingly, in the two invasive CC samples of our cohort, the integration of HPV16 is weak which supports the current controversy about the role of HPV integration in the onset and progression of CC. In addition, although the integration of the HPV genome has long been considered a late event in cervical oncogenesis, recent controversial results showing early detection of HPV integration and poor performance of this biomarker suggest a more complex interplay [27,42].

Our data clearly demonstrate that E6/E7 mRNA VLs in UCS represent useful biomarkers to identify women harboring high grade cervical lesions. Indeed, we showed that expression of E6/E7 mRNA is significantly increased in UCS obtained from women developing CIN2+ and CIN3+ lesions. We performed E6/E7 mRNA VL quantification using an in-house qRT-PCR assay involving three primer/probe sets designed to amplify various E6/E7 mRNA isoforms. Our results indicate that the use of any of the three sets allows proper identification of CIN2+ and CIN3+ lesions, with the SP-E6/E7 set exhibiting the best diagnostic performance (Table 6). This result was expected since SP-E6/E7 set quantifies all RNA isoforms coding for E6 and E7 proteins, which are the main contributors to HPV-driven carcinogenesis [43]. Interestingly, E6^E7 VL, reflecting the E6/E7 mRNA with disrupted E6 and E7 ORFs, also appear to correlate with high grade lesions. This suggests that E6^E7mRNA derived from splicing of the HPV16 pre-mRNA may contribute to the development of cervical lesions. This conclusion is reinforced by the observation that one patient in our cohort harbors a CIN2 lesion (patient 4) while only E6^E7 mRNA are detectable in her UCS. Although the mechanism involved in this process needs to be elucidated, one possibility might be that E6^E7 mRNA improve the stability of E6 and E7 oncoproteins through the production of an E6^E7 fusion protein [31]. In two CIN1 patients (patients 42 and 51), quantification of E6/E7 mRNA VLs using the SP-E6/E7 set resulted in an undetectable VL, while E6/E7 mRNA were amplified by the FL-E6/E7 set (Table 4).

PCR amplification using the FL-E6/E7 set would have result in co-amplification of both sets (Figure 1) as expected and previously described by Ajiro et al. [29]. This unexpected result might be explained by the existence of additional, yet undescribed, E6/E7 mRNA isoforms containing intron 1 but lacking sequences targeted by the SP-E6/E7 set. Interestingly, no amplification was seen in sample 43 using the FL-E6/E7 set while amplification using the SP-E6/E7 set gave a positive result. As patient 43 exhibits a CIN1 grade histological lesion, this indicates that the expression of E7 alone might be sufficient to promote initial steps of cervical dysplasia.

Our results demonstrate that the expression of all E6/E7 mRNA isoforms is increased in high grade cervical lesions compared to low grade lesions, which suggest that each of the various E6/E7 mRNA species may contribute to CC progression (Table 5). These data warrant further investigations, as little is known on the biological functions of E6/E7 mRNA isoforms. E6*I is abundantly expressed in CC cell lines and UCS and seems to support E7 expression [44,45,46]. The E6*I truncated protein translated from the E6*I mRNA has been shown to exert both pro- and anti-tumorigenic functions [30,47]. The E6^E7 fusion protein expressed from the E6^E7 isoform may favor oncogenic activities of E6 and E7 proteins, while biological activities of E6*II, E6*V and E6*VI have not been investigated yet [31].

Analysis of diagnostic performances in our cohort shows that SP-E6/E7 VL quantification by qRT-PCR is an efficient assay for CC screening. All parameters commonly used to assess the quality and the validity of diagnostic tests, such as Youden index, sensitivity, specificity, NPV, PPV, and AUC derived from ROC analysis, exhibited values supporting the relevance of this assay for proper triage of patients harboring high grade cervical lesions. Interestingly, sensitivity and specificity are better balanced for SP-E6/E7 VL than for the Pap test regarding the detection of CIN2+ lesions. It is desirable to reach an optimal equilibrium between these two parameters as a good sensitivity prevents misidentification of diseased patients, while a high specificity avoids over-management of healthy women. In our study, the use of SP-E6/E7 VL in first intention would have allowed proper identification of CIN2+ patients undiagnosed by the cytological approach. Indeed, the sensitivity obtained with the SP-E6/E7 VL set was higher (90% (56–100) than the one obtained with the Pap test (80% (44–97)), while specificity was slightly lower (90% (56–100) vs. 100% (69–100)).

In contrast, HPV16 DNA quantification in UCS does not appear to be relevant for identification of CIN2+ lesions, as DNA VLs are not significantly increased in CIN2+ patients compared to <CIN2 patients. In addition, diagnostic performances of DNA VLs are lower than that of the SP-E6/E7 VL and Pap test. As DNA detection tests, the use of DNA VL assays might be limited as they allow detection of HPV infection and quantification of HPV replication without bringing any information on the transcriptional status of HPV oncogenic genes. Indeed, HPV infections associated with both high level of viral replication and absence or low oncogenic E6 and E7 proteins expression might not represent a risk for cervical neoplasia and cancer progression.

More importantly, our data showed that SP-E6/E7 mRNA VL might represent a biomarker with an interesting diagnostic added value and an increased accuracy to complement Pap test which could predict histological severity grade for ASC-US and LSIL patients. We were able to successfully classify two out of three ASC-US patients (patients 45 and 55) as <CIN2 patients, based on SP-E6/E7 VL values (2.49 and 3.04 log10 copies/10^6^ β-actin mRNA copies, respectively) which were below the cut-off threshold set (3.16 log10 copies/10^6^ β-actin mRNA copies). The third ASC-US patient (patient 4) was not correctly classified as a CIN2+ patient using SP-E6/E7 VL values, but this sample exhibited a specific profile as we detected low DNA VLs and only E6^E7 mRNA. We thus hypothesized that the UCS may have been collected from a healthy cervical region, outside the area of the lesion. This is a limitation of this study: cervical sampling performed as part of CC screening is performed blind. This may explain why DNA and E6/E7 mRNA VLs are below the values expected for a high grade CIN2+ lesion. Regarding the other E6/E7 mRNA VLs (FL-E6/E7, T-E6/E7 and E6^E7), the values obtained allowed a proper classification of 1 out of 3 ASC-US patients only. This observation supports the conclusion that additional quantification of E6/E7 mRNA spliced isoforms retaining an intact E7 ORF in UCS allows the detection of high grade cervical lesions with a better accuracy than the quantification of unspliced E6/E7 mRNA by itself. In addition, we managed to identify the histological severity grade of all LSIL patients (9, 15, 18, and 51) using the SP-E6/E7 mRNA VL biomarker. Parallel to the good performances and prospects of clinical application of our test, we must take into account some inherent disadvantages in terms of technique and cost for a city medical analysis laboratory. Indeed, the extraction of mRNA from smears is a delicate step: It is necessary to use an RNA extraction method adapted to short RNAs (<200 bp). This is a time-consuming manual method which will impact the turnaround time compared to current DNA approaches. Moreover, this method is also expensive in terms of extraction reagent, qPCR equipment, and trained personnel required in molecular biology. Finally, our method is initially designed for monoinfections by HPV16. Additional studies are needed to expand use to other HPV-like infections, including HPV 18 and multiple infections.

These data emphasize that SP E6/E7 mRNA VL quantification tests are much more relevant as first-intention tests than cytology and represent an added value to the standard cytological analysis currently used, especially in cases of equivocal (ASC-US) or low-grade (LSIL) cytological lesions. This newly improved diagnostic method could benefit the CC screening. Further investigations remain necessary to confirm these results on a larger and prospective cohort in order to improve clinical diagnosis.

## Figures and Tables

**Table 1:** Sequence-specific oligonucleotide primers and probes used for quantification of human papillomavirus (HPV) 16 DNA viral load (VL) by quantitative polymerase chain reaction (qPCR).

Primer/Probe Set		Sequences
HPV16 E6	Forward	5′-GAGAACTGCAATGTTTCAGGACC-3′
	Reverse	5′- TGTATAGTTGTTTGCAGCTCTGTGC-3′
	Probe	JOE-5′-CAGGAGCGACCCAGAAAGTTACCACAGTT-3′-TAMRA
HPV16 E2	Forward	5′-AACGAAGTATCCTCTCCTGAAATTATTAG-3′
	Reverse	5′-CCAAGGCGACGGCTTTG-3′
	Probe	FAM-5′-CACCCCGCCGCGACCCATA-3′-TAMRA
β-globin	Forward	5′-TGCATCTGACTCCTGAGGAGAA-3′
	Reverse	5′-GGGCCTCACCACCAACTTC-3′
	Probe	TET-5′-CTGCCGTTACTGCCCT-3′-TAMRA

**Table 2:** Sequence-specific oligonucleotide primers and probes used for quantification of HPV16 E6/E7 mRNA VL by quantitative reverse transcription polymerase chain reaction (qRT-PCR).

Primer/Probe Set		Sequences
FL-E6/E7	Forward	5′-GTGTACTGCAAGCAACAGTTA-3′
	Reverse	5′-CCCATCTCTATATACTATGCATAAATCC-3′
	Probe	FAM-5′-CTGCGACGTGAGGTATATGACTTTGCT-3′-TAMRA
SP-E6/E7	Forward	5′-GATTTGCAACCAGAGACAACTG-3′
	Reverse	5′-GCTGGACCATCTATTTCATCCT-3′
	Probe	FAM-5′-TGAGCAATTAAATGACAGCTCAGAGGAGG-3′-TAMRA
T-E6/E7	Forward	5′-GACTCTACGCTTCGGTTGTG-3′
	Reverse	5′-TGTGCCCATTAACAGGTCTT-3′
	Probe	FAM-5′-CGTACAAAGCACACACGTAGACATTCG-3′-TAMRA
β-actin	Forward	5′-GACCCAGATCATGTTTGAGACC-3′
	Reverse	5′-CCAGAGGCGTACAGGGATA-3′
	Probe	FAM-5′-TGTACGTTGCTATCCAGGCTGTGC-3′-TAMRA

**Table 3:** Quantification of HPV16 DNA and E6/E7 mRNA VLs in SiHa cells.

Viral Load Type		DNA VL (Copies/Cell ± SD (Range))	E6/E7 mRNA VL (log_10_ (Copies/10^6^ β-actin mRNA Copies) ±SD (Range))
DNA	Total	0.93 ± 0.2 (0.7–1.06)	
	Episomal	0	
	Integrated	0.93 ± 0.2 (0.7–1.06)	
mRNA	FL-E6/E7		4.47 ± 0.18 (4.27–4.59)
	SP-E6/E7		4.86 ± 0.21 (4.63–5.00)
	T-E6/E7		4.93 ± 0.23 (4.68-5.12)
	E6^E7		4.09 ± 0.37 (3.73-4.48)

SD, standard deviation; VL, viral load; FL, full length E6/E7 mRNA; SP, FL + spliced E6/E7 mRNA containing intact E7 ORF; T, Total E6/E7 mRNA corresponding to SP + E6^E7 mRNA; E6^E7, E6/E7 mRNA containing disrupted E6 and E7 ORFs, calculated by the following subtraction T-SP.

**Table 4:** Quantification of HPV16 DNA and E6/E7 mRNA VLs in uterine cervical smears (UCS).

Patient ID	Cytology Grade	Histology Grade	HPV16 DNA VL (log10 (Copies/10^6^ Cells))			HPV16 mRNA VL (log10 (Copies/10^6^ β-actin mRNA Copies))			
			Total	Episomal	Integrated	FL-E6/E7	SP-E6/E7	T-E6/E7	E6^E7
31	Normal	Unlesional	5.92	5.62	5.61	2.02	2.34	2.86	2.71
44	Normal	Unlesional	6.23	5.77	6.04	0.00	0.00	2.90	2.90
15	LSIL	CIN1	6.80	6.01	6.73	2.15	2.73	3.99	3.97
18	LSIL	CIN1	7.56	6.86	7.46	1.78	2.15	3.50	3.48
34	Normal	CIN1	6.08	6.06	4.81	3.45	4.06	4.09	2.94
42	Normal	CIN1	5.35	5.06	5.03	1.76	0.00	3.31	3.31
43	Normal	CIN1	6.59	6.35	6.22	0.00	1.59	2.95	2.93
45	ASC-US	CIN1	7.61	6.76	7.55	1.80	2.49	4.34	4.34
51	LSIL	CIN1	6.95	6.36	6.83	2.97	0.00	4.30	4.30
55	ASC-US	CIN1	5.89	5.79	5.19	2.91	3.04	3.27	2.87
2	ASC-H	CIN2	6.97	6.17	6.89	2.80	3.16	3.88	3.79
4	ASC-US	CIN2	5.37	4.71	5.27	0.00	0.00	1.91	1.91
9	LSIL	CIN2	7.83	7.09	7.75	3.05	3.79	5.01	4.99
47	ASC-H	CIN2	6.96	6.47	6.79	3.53	3.94	4.45	4.29
1	HSIL	CIN3	8.09	7.48	7.97	3.52	4.19	4.72	4.56
5	ASC-H	CIN3	7.19	6.55	7.08	4.25	4.81	5.10	4.79
41	HSIL	CIN3	5.51	5.12	5.28	2.54	3.17	3.51	3.23
50	ASC-H	CIN3	7.63	6.94	7.52	4.12	5.16	5.62	5.44
3	HSIL	Invasive Cancer	7.88	7.67	7.47	3.91	4.49	5.67	5.64
6	HSIL	Invasive Cancer	7.97	7.62	7.72	4.11	4.81	5.26	5.07

LSIL, low-grade squamous intraepithelial lesion; ASC-US, atypical squamous cells of unknown significance; ASC-H, atypical squamous cells- cannot exclude high grade; HSIL, high grade squamous intraepithelial lesion; CIN, cervical intraepithelial neoplasia; VL, viral load; FL, full length E6/E7 mRNA; SP, FL + spliced E6/E7 mRNA containing intact E7 ORF; T, Total E6/E7 mRNA corresponding to SP + E6^E7 mRNA; E6^E7, E6/E7 mRNA containing disrupted E6 and E7 ORFs, calculated by the following subtraction T-SP.

**Table 5:** Statistical analysis of HPV16 DNA and E6/E7 mRNA VLs variations in high grade cervical lesions.

HPV16 Viral Load Type			CIN2+ Histology Threshold			CIN3+ Histology Threshold		
			Histology <CIN2	Histology CIN2+	*p*-Value (Wilcoxon)	Histology <CIN3	Histology CIN3+	*p*-Value (Wilcoxon)
DNA VL (log10 (copies/10^6^ cells))	Total	Mean ± SD	6.5 ± 0.7	7.1 ± 1.0	0.0690	6.6±0.8	7.4 ± 1.0	**0.0490**
	Integrated	Mean ± SD	6.1 ± 1	7.0 ± 1.0	0.0596	6.3±1.0	7.2 ± 1.0	0.0676
E6/E7 mRNA VL (log10 (copies/10^6^ β-actin mRNA copies))	FL-E6/E7	Mean ± SD	1.9 ± 1.2	3.2 ± 1.3	**0.0201**	2.0±1.2	3.7 ± 0.6	**0.0102**
	SP-E6/E7	Mean ± SD	1.8 ± 1.4	3.8 ± 1.5	**0.0112**	2.1±1.5	4.4 ± 0.7	**0.0048**
	T-E6/E7	Mean ± SD	3.6 ± 0.6	4.5 ± 1.2	**0.0279**	3.6±0.8	5.0 ± 0.8	**0.0124**
	E6^E7	Mean ± SD	3.4 ± 0.6	4.4 ± 1.1	**0.0380**	3.5±0.8	4.8 ± 0.9	**0.0177**

CIN, cervical intraepithelial neoplasia; VL, viral load; SD, standard deviation; FL, full length E6/E7 mRNA; SP, FL + spliced E6/E7 mRNA containing intact E7 ORF; T, total E6/E7 mRNA corresponding to SP + E6^E7 mRNA; E6^E7, E6/E7 mRNA containing disrupted E6 and E7 ORFs is calculated by the following subtraction T-SP; *p*-value ≤ 0.05 for the highlighted statistics.

**Table 6:** Diagnostic performances of the Pap test. HPV16 DNA and E6/E7 mRNA VLs in detection of high grade cervical lesions.

HPV16 Viral Load Type		CIN2+ Histology Threshold							CIN3+ Histology Threshold						
		TV	YI	NPV. % (95%CI)	PPV. % (95%CI)	Se.% (95%CI)	Spe. % (95%CI)	AUC (95%CI)	TV	YI	NPV. % (95%CI)	PPV. % (95%CI)	Se.% (95%CI)	Spe. % (95%CI)	AUC (95%CI)
DNA	Total	6.96	0.60	80 (44–97)	80 (44–97)	80 (44–97)	80 (44–97)	0.76 (0.52–1.00)	7.19	0.62	92 (36–100)	63 (24–91)	83 (36–100)	79 (49–95)	0.81 (0.53–1.00)
	Integrated	6.79	0.50	78 (40–97)	73 (39–94)	80 (44–97)	70 (35–93)	0.77 (0.55–0.99)	7.08	0.62	92 (62–100)	63 (24–91)	83 (36–100)	79 (49–95)	0.79 (0.54–1.00)
mRNA	FL-E6/E7	2.54	0.60	88 (47–100)	75 (43–95)	90 (56–100)	70 (35–99)	0.84 (0.65–1.00)	2.54	0.57	100 (63–100)	50 (21–79)	100 (54–100)	57 (29–82)	0.92 (0.77–1.00)
	SP-E6/E7	3.16	0.80	90 (56–100)	90 (56–100)	90 (56–100)	90 (56–100)	0.88 (0.69–1.00)	3.17	0.79	100 (72–100)	67 (30–93)	100 (54–100)	79 (49–95)	0.97 (0.89–1.00)
	T-E6/E7	3.51	0.50	86 (42–100)	69 (39–91)	90 (56–100)	60 (26–88)	0.82 (0.60–1.00)	4.72	0.76	93 (66–100)	83 (36–100)	83 (36–100)	93 (66–100)	0.91 (0.73–1.00)
	E6^E7	3.23	0.40	83 (36–100)	64 (35–87)	90 (56–100)	50 (19–81)	0.80 (0.58–1.00)	3.23	0.43	100 (54–100)	43 (18–71)	100 (54–100)	43 (18–71)	0.88 (0.69–1.00)
Pap test		NA	NA	83 (52–98)	100 (63–100)	80 (44–97)	100 (69–100)	NA	NA	NA	100 (74–100)	75 (35–97)	100 (54–100)	86 (57–98)	NA

TV, threshold value; YI, Youden index; NPV, negative predictive value; PPV, positive predictive value; Se, sensitivity; Spe, specificity; CIN, cervical intraepithelial neoplasia; AUC, area under roc curve.

## Abbreviations

HPVs	human papillomaviruses
FL	full length E6/E7 mRNA
SP	+ spliced E6/E7 mRNA containing intact E7 ORF
T	total E6/E7 mRNA corresponding to SP + E6^E7 mRNA
VL	viral loads
E6^E7	E6/E7 mRNA containing disrupted E6 and E7 ORFs calculated by the following subtraction T-SP
ASC-US	atypical squamous cells of unknown significance
LSIL	low-grade squamous intraepithelial lesion
CC	cervical cancer
CIN	cervical intraepithelial neoplasia
CIN2+	CIN of grade 2 or more: CIN2, CIN3, cancer
CIN3+	CIN of grade 3 or more: CIN3, cancer
UCS	uterine cervical smears
ASC-H	atypical squamous cells- cannot exclude high grade
HSIL	high grade squamous intraepithelial lesion
ROC	receiver operating curves
AUC	area under roc curve
NPV	negative predictive value
PPV	positive predictive value
